# The Use of Light‐Based Therapies in the Treatment of Alopecia

**DOI:** 10.1111/jocd.70434

**Published:** 2025-08-29

**Authors:** Robert J. Vanaria, Aysham Chaudry, Mark S. Nestor

**Affiliations:** ^1^ Center for Clinical and Cosmetic Research Aventura Florida USA; ^2^ Hackensack Meridian School of Medicine Nutley New Jersey USA; ^3^ Department of Dermatology and Cutaneous Surgery University of Miami Miller School of Medicine Miami Miami Florida USA; ^4^ Division of Plastic Surgery, Department of Surgery University of Miami Miller School of Medicine Miami Miami Florida USA

**Keywords:** alopecia, hair loss, low level light therapy, photobiomodulation

## Abstract

**Background:**

Alopecia encompasses a group of conditions that can significantly impact quality of life, especially among women and younger individuals. While pharmaceutical therapies remain the cornerstone of treatment, laser and light‐based therapies, especially low‐level light therapy (LLLT), offer promising noninvasive alternatives. LLLT uses specific wavelengths of light to stimulate hair follicle repair, prolong the hair growth phase, and promote regrowth. Recent advancements in at‐home devices and dual‐wavelength LED systems have expanded access to these therapies. This review explores the role of LLLT in treating alopecias, evaluating its mechanisms, efficacy, and clinical applications.

**Methods:**

A PubMed search using terms related to alopecia and laser/light therapy was conducted. Results were limited to English‐only articles from 2020 to 2025, excluding duplicates. Additional articles were identified through citation tracking. Studies not focused on light‐based therapies were excluded.

**Results:**

Of 403 articles identified, 63 were included based on relevance to alopecia or hair loss and light‐based therapy. Results were categorized by treatment modality and type of hair loss, with overlap between categories.

**Discussion:**

This review highlights the growing role of LLLT as an adjunct or alternative treatment for various types of alopecia. In androgenetic alopecia, LLLT improves hair density and follicular responsiveness, with enhanced outcomes when combined with minoxidil or finasteride. For telogen effluvium, LLLT shows potential in prolonging the anagen phase and reducing shedding, although larger studies are needed. In alopecia areata, LLLT may promote regrowth by modulating immune responses and improving perifollicular microcirculation. Emerging data also support LLLT in lichen planopilaris and central centrifugal alopecia (CCCA), with case reports showing reduced inflammation and hair regrowth. Overall, LLLT offers a noninvasive, well‐tolerated option across alopecia subtypes, though standardized protocols and long‐term data remain limited.

## 1 Introduction

Alopecia refers to a group of multifactorial conditions that affect millions of patients worldwide, with various subtypes distinguished by their underlying etiology, clinical presentation, and progression. It is broadly classified into scarring and non‐scarring alopecias [1]. Its prevalence varies by subtype and population, with androgenetic alopecia (AGA) affecting up to 50% of men and 30%–40% of women by age 50 and alopecia areata (AA) affecting approximately 2% of the general population [2, 3].

Alopecia can have significant psychological and social impacts, and in the case of scarring alopecia, significant morbidity, making effective treatment crucial. The psychological burden is most notable among younger individuals and women, where hair loss can severely affect self‐esteem and quality of life [4, 5]. While pharmaceutical management for alopecia has been the mainstay of treatment, laser and light‐based therapies have emerged as promising, noninvasive treatment modalities [4].

Laser and light‐based therapies, particularly low‐level light therapy (LLLT) or photobiomodulation, offer a novel therapeutic avenue that continues to evolve with ongoing technological advancements and clinical research of the various types of alopecia. It is referred to as “low level” because it delivers a lower amount of energy than other light‐based devices, typically within the range of 1–9 J/cm^2^ [6]. Photobiomodulation involves the use of specific wavelengths of low level light (typically in the red or near‐infrared spectrum) to stimulate cellular processes and enhance tissue repair, including within hair follicles [7]. These therapies are believed to prolong the anagen phase of the hair cycle, increase vascularization around the follicles, and promote the proliferation of dermal papilla cells, thereby encouraging hair regrowth and reducing hair thinning [8]. LLLT at‐home devices such as combs, helmets, and caps have shown varying degrees of positive results in clinical studies for alopecia, with side effects primarily limited to mild scalp irritation and pruritus and overall good patient tolerability [9, 10, 11]. Additionally, recent technological advancements have led to more efficient light delivery systems and home‐use devices that expand access and convenience for patients, particularly regarding dual wavelength LED devices [10]. Ongoing clinical trials and mechanistic research continue to refine our understanding of optimal wavelengths, treatment regimens, and combinations with other therapies, positioning light‐based treatments and photobiomodulation as a valuable component in the management of hair loss [12]. This review explores the role of LLLT in managing different alopecias, examining their mechanisms, efficacy, and clinical applications.

## 2 Materials and Methods

A PubMed search was conducted using the terms “alopecia” OR “hair loss” AND “laser” OR “photodynamic therapy” OR “light”. Results were screened to include English‐only articles between 2020 and 2025 and excluded duplicates. Results were then screened for relevance and excluded articles that did not focus on laser/light therapy for treatment of alopecias or hair loss specifically. Further exploration of laser and light therapy as a therapy for alopecias was enhanced through citation tracking and additional PubMed searches. This resulted in the inclusion of some articles from dates outside of our searched window.

## 3 Results

**FIGURE 1:**
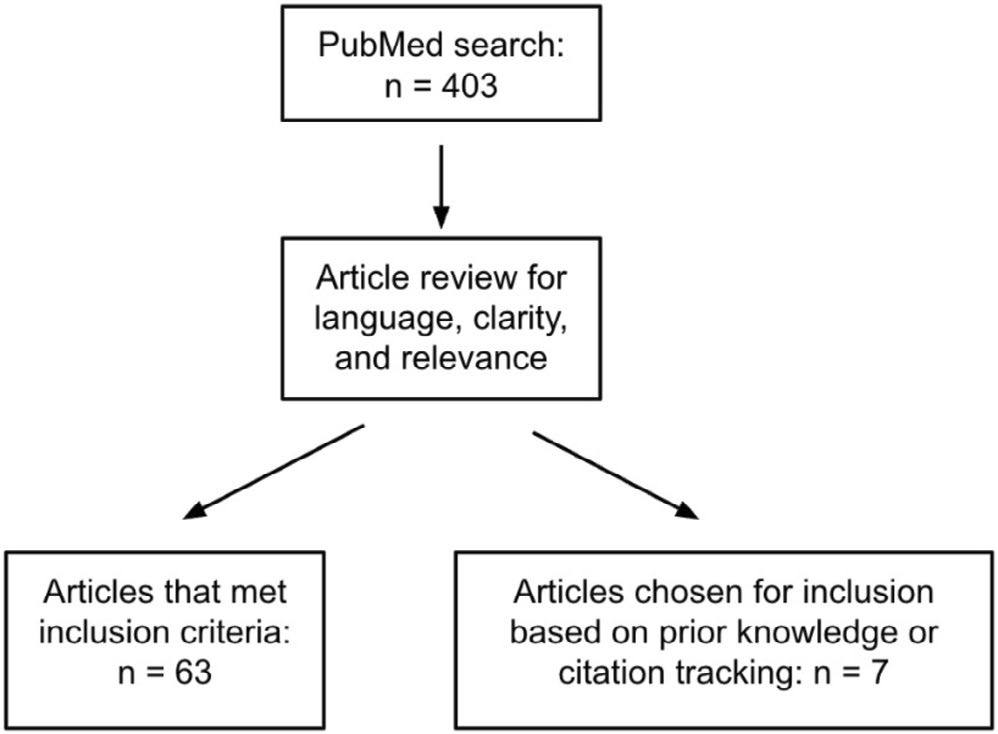
Flowchart depicting PubMed search strategy and article inclusion.

A total of 403 articles resulted from our search criteria. Of that, 63 articles were chosen for inclusion in this review based on their relevance to both alopecias and hair loss and light therapy as a treatment modality. Results were categorized based on their treatment modality and type of hair loss treated and reported in the appropriate subsection. Inclusion in one of the categories did not preclude data inclusion in another. A flowchart is included below illustrating the search process for articles included in our review (Figure 1).

## 4 Discussion

For the purposes of this review, the discussion section is organized by diagnosis with commentary and results following. AGA and telogen effluvium (TE) were chosen as the first two sections due to their prevalence and often clinically indistinguishable presentations.

### 4.1 Androgenetic Alopecia

AGA is the most common form of hair loss, affecting up to 80% of men and nearly 50% of women over the course of their lives [13]. In males, AGA typically presents as progressive hair loss in the vertex, bitemporal, or midfrontal scalp. In females, the presentation is often more diffuse, with thinning concentrated along the central scalp while generally sparing the frontal hairline [2, 4].

The pathophysiology of AGA centers around the androgen dihydrotestosterone (DHT), a potent metabolite of testosterone. DHT is produced via the enzymatic conversion of testosterone by 5α‐reductase (5aR). Once formed, DHT binds to androgen receptors on hair follicles, particularly in androgen‐sensitive areas of the scalp, initiating a cascade that shortens the anagen (growth) phase and prolongs the telogen (resting) phase of the hair cycle [14, 15]. This results in progressive miniaturization of terminal hair follicles, transforming thick, pigmented terminal hairs to short, fine, vellus hairs over time [4, 16].

Diagnosis of AGA is primarily clinical, based on characteristic patterns of hair loss observed during physical examination. In males, the Norwood–Hamilton classification system is commonly used to stage the severity of hair loss, whereas in females, the Ludwig scale is the standard [17, 18].

Conventional treatments for AGA include topical arteriolar dilators such as minoxidil to increase blood flow and potentially prolong the anagen phase, or oral 5aR inhibitors such as finasteride or dutasteride, which reduce DHT levels and mitigate follicular miniaturization [19]. Additionally, nutrient supplements have shown some efficacy as well as platelet‐rich plasma [4, 20]. Despite these therapies, limitations such as side effects, variable response, and poor long‐term adherence have led to growing interest in alternative and adjunctive approaches, including LLLT.

LLLT has emerged as a promising treatment modality for AGA. In‐office and at‐home devices typically use red (wavelengths between 620 and 680 nm), which penetrate the scalp and stimulate follicular cells. The proposed mechanisms include increased ATP production in mitochondria, upregulation of antiapoptotic proteins, improved microcirculation, and modulation of inflammatory mediators in the hair follicle environment [7, 21].

Several clinical studies have supported the efficacy of LLLT in both male and female AGA. In a double‐blind, placebo device‐controlled trial by Leavitt et al. participants using an LLLT device demonstrated a significant increase in terminal hair density compared to the placebo device after 26 weeks [22]. A dual wavelength LED LLLT device (Revian System, Revian Inc.) has been shown to decrease DHT production via stimulated nitric oxide production within cells specific to hair growth [23, 24]. Results from a prospective, randomized‐control, double‐blind, parallel study to evaluate the efficacy and safety of this all‐LED, dual wavelength red light therapy device in men and women with AGA revealed that subjects who were treated with the dual wavelength LED device and were at least 80% compliant for the duration of the study had an average of 21 more hairs per cm^2^ compared to those who used a placebo device after 16 weeks. Subjects treated with placebo continued to lose hair over the duration of the study [10]. Another study looking at the dual wavelength LED device also demonstrated strong compliance (80%), indicating both ease of use and efficacy [25]. Similarly, Kim et al. showed that both men and women treated with LLLT helmets experienced statistically significant improvements in hair counts and subjective assessments of hair thickness and satisfaction [11]. These findings suggest that LLLT can not only slow the progression of hair loss but also may promote regrowth in affected individuals.

Combining LLLT with established treatments such as minoxidil or finasteride has been shown to potentially enhance clinical outcomes compared to monotherapy. In a randomized control trial by Lanzafame et al. participants using both LLLT and 5% topical minoxidil demonstrated significantly greater increases in hair density than those using either therapy alone, suggesting a synergistic effect [26]. Similarly, a study by Munck et al. found that combining finasteride with LLLT led to improved hair growth parameters, including hair count and patient satisfaction, when compared to finasteride alone [27]. These findings support the hypothesis that photobiomodulation may potentiate the effects of pharmacologic agents by improving follicular responsiveness, enhancing blood flow, and mitigating inflammation. As such, combination therapy may be particularly beneficial for individuals with moderate to severe AGA or for those seeking more robust results within a shorter timeframe.

LLLT is generally well‐tolerated with minimal reported side effects, the most common being transient, self‐resolving scalp pruritus. Furthermore, the availability of at‐home devices has improved accessibility and convenience for patients, and dual wavelength devices linked to a phone‐based app can significantly improve compliance. While results may vary depending on treatment adherence, device quality, and baseline severity, LLLT, particularly dual wavelength devices, represents a valuable addition to the therapeutic arsenal for AGA, particularly when used in combination with established pharmacologic treatments.

### 4.2 Telogen Effluvium

TE is a non‐scarring, diffuse alopecia that results from a disruption of the hair cycle, specifically a premature shift of hair follicles from the anagen (growth) phase to telogen (resting) phase. Common triggers include emotional stress, systemic illness, nutritional deficiencies, medications, childbirth, and hormonal changes [28]. TE typically presents with excessive daily hair shedding, often with a positive hair pull test, and it is more prevalent in women.

Diagnosis is clinical but can be supported by dermoscopy or trichoscopy, which may reveal empty follicular ostia and a predominance of telogen hairs. Acute TE generally resolves spontaneously within 6 months if the trigger is removed, while chronic TE persists beyond 6 months and may require targeted treatment.

Conventional management of TE includes addressing underlying causes, nutritional optimization, and in some cases, topical minoxidil [29]. However, due to patient frustration and variability in recovery, adjunctive therapies like LLLT have gained attention.

LLLT enhances mitochondrial activity, increases ATP production, and prolongs the anagen phase, directly addressing the underlying cause of TE. In a study by Amer et al. seven women with clinically diagnosed TE were treated with a LLLT device twice weekly for 16 weeks. While the improvement in total hair count was not statistically significant (mean increase of 8.7%; *p*‐value = 0.143), patients reported subjective benefits in hair shedding and density. Notably, 71.4% of patients reportedly improved hair density, and 57.1% experienced less hair fall, with no serious adverse effects [30]. Similarly, Sorbellini et al. reported that LED‐based LLLT may support recovery in stress‐induced and reactive TE by enhancing vascular and metabolic support to follicles. The therapy was proposed as a useful noninvasive adjunct, particularly for chronic or relapsing TE, although more robust studies are needed to confirm long‐term outcomes [31].

### 4.3 Alopecia Areata

AA is an autoimmune disorder characterized by non‐scarring, patchy hair loss, most commonly affecting the scalp, although any hair‐bearing area can be involved. Hair loss may be present in distinct patterns, including the ophiasis pattern (localized to the occipital and temporal regions), sisaipho (reverse ophiasis, involving the frontal and parietal scalp), and diffuse pattern. In severe cases, AA can progress to alopecia totalis (loss of all scalp hair) or alopecia universalis (loss of all body hair) [32, 33].

The underlying pathogenesis of AA involves a T‐cell‐mediated immune response directed against hair follicles during the anagen phase. Histopathology typically reveals a “swarm of bees” lymphocytic infiltrate comprised primarily of CD8+ cytotoxic T‐cells and CD4+ helper T‐cells [34]. Additionally, antibodies targeting hair follicle antigens, including IgG subclasses, and elevated interferon gamma have been implicated, suggesting a role for both cellular and humoral immunity [35]. AA is considered a polygenic disease, with genome‐wide association studies identifying multiple susceptibility loci linked to immune function, including HLA‐DR, CTLA‐4, and IL2‐Rα [36]. AA can also coexist with other autoimmune conditions, including thyroid disease, vitiligo, and atopic dermatitis [37].

While corticosteroids (topical, intralesional, or systemic) remain a mainstay of therapy, and the recent introduction of Janus Kinase (JAK) inhibitors such as ritlecitinib has shown tremendous promise for severe AA, a growing body of research supports the role of light‐based treatments (including LLLT) in AA and has been explored as adjunctive or alternative options to immunosuppressive agents [38, 39].

Single wavelength devices, such as the 308 nm excimer laser, have shown beneficial outcomes in the treatment of AA. This modality is believed to induce T‐cell apoptosis and suppress local cytokine signaling. Multiple studies have demonstrated partial to complete regrowth in treated patches, with relatively few adverse effects [40]. In a study by Al‐Mutairi from 2007, 41.5% of total patches of AA treated experienced at least 50% regrowth of hair, and thirteen of eighteen scalp lesions (72.2%) showed complete regrowth of hair after 12 weeks. Notably, this study demonstrated that extremity regions failed to show response to laser treatment, suggesting a potential advantage for application in more centrally located AA lesions [41].

LLLT has been proposed to modulate immune responses in AA through anti‐inflammatory and immunoregulatory mechanisms. While evidence remains limited, case series and pilot studies suggest that LLLT can promote hair regrowth by reducing perifollicular inflammation and enhancing microcirculation. Patients with AA were able to see significant increases in both hair assessment scales and in manual hair counting after twice weekly LLLT treatments for 2 months [42]. This is partly attributed to the ability of LLLT to activate cytochrome C in mitochondria, increasing electron transport and therefore ATP. This reverts hair follicles out of the dormant, telogen phase and into the growth, anagen phase [26]. Additional proposed mechanisms include additional, unrecognized pathways involved in the pathogenesis of AA including those on the subcellular and molecular level that LLLT is able to reduce or reverse completely [43].

### 4.4 Lichen Planopilaris

Lichen planopilaris (LPP) is a primary, lymphocytic cicatricial alopecia characterized by perifollicular inflammation leading to permanent hair loss. Clinically, LPP presents with multifocal patches of alopecia, perifollicular erythema, scaling, and follicular hyperkeratosis, predominantly affecting the vertex and parietal scalp regions [44]. Histopathologically, LPP exhibits a lichenoid interface dermatitis with a perifollicular lymphocytic infiltrate, basal vacuolar degeneration, and concentric perifollicular fibrosis, particularly at the level of the isthmus and infundibulum [45].

The pathogenesis of LPP is not fully understood, but it is believed to involve an autoimmune response targeting follicular antigens, leading to follicular destruction and scarring. Traditional treatments aim to halt disease progression and alleviate symptoms. First‐line treatment options include high‐potency topical corticosteroids and topical calcineurin inhibitors. In more extensive or refractory cases, systemic agents such as hydroxychloroquine, cyclosporine, and mycophenolate mofetil have been employed, with recent data on JAK inhibitors also emerging [46, 47, 48, 49, 50].

LLLT has also emerged as a potential adjunctive treatment for LPP. A case series reported four patients with LPP who demonstrated significant improvement following LLLT, including a dramatic reduction in inflammation, symptom resolution, and visible hair regrowth without adverse effects [51]. These results indicate a route for further exploration of LLLT as a desirable treatment option for LPP and other lichenoid conditions. Larger, controlled studies should be conducted to establish the efficacy and safety of LLLT in the management of LPP.

### 4.5 Frontal Fibrosing Alopecia

Frontal fibrosing alopecia (FFA) is a primary lymphocytic cicatricial alopecia and a clinical variant of LPP. It is characterized by progressive recession of the frontotemporal hairline, often accompanied by eyebrow loss, perifollicular erythema, and follicular hyperkeratosis [52]. FFA primarily affects postmenopausal women but can also occur in premenopausal women and men [52, 53]. The pathogenesis of FFA is not fully understood, but it is believed to involve immune‐mediated destruction of follicular epithelial stem cells in the bulge region of the hair follicle unit, possibly triggered by hormonal or environmental factors. Histologically, it is marked by perifollicular lymphocytic inflammation and fibrosis, leading to permanent follicle loss [54].

Traditional treatments for FFA have included topical and intralesional corticosteroids, calcineurin inhibitors, hydroxychloroquine, 5aR inhibitors, and systemic immunosuppressants such as mycophenolate mofetil and methotrexate. These therapies aim to reduce inflammation and slow disease progression, though hair regrowth is rarely achieved once fibrosis has occurred [55]. New data has also emerged on the use of both topical and oral JAK inhibitors for the treatment of FFA; however, the studies are limited by sample size and quantity and therefore need further exploration [48, 49, 50]. Given the complex nature of FFA, light‐based therapies have not historically been a first‐line treatment. However, recent interest has emerged in LLLT as a potential adjunctive option due to its ability to stimulate follicular stem cell activity, enhance mitochondrial function, and exert anti‐inflammatory effects, which may benefit early‐stage FFA where active inflammation precedes irreversible fibrosis [7, 26]. Although no trials or case reports have demonstrated efficacy in treating FFA specifically, the existing data on LLLT for LPP is encouraging for future studies on LLLT for FFA.

### 4.6 Central Centrifugal Cicatricial Alopecia

Central centrifugal cicatricial alopecia (CCCA) is the most common form of scarring alopecia in women of African descent and is characterized by progressive hair loss that typically begins at the crown and spreads centrifugally [56]. The clinical presentation includes decreased hair density, perifollicular scaling, and follicular dropout. Histopathological features show follicular and infundibular hyperkeratosis, perifollicular lymphocytic infiltration, and concentric fibrosis around the follicle, which leads to permanent hair loss if untreated [57].

The exact etiology of CCCA remains unclear, but contributing factors may include genetic predisposition, inflammation, and hair grooming practices that induce chronic mechanical or thermal trauma [58]. Mutations in the PADI3, involved in hair shaft formation, have been implicated in familial cases [58].

Current treatment strategies focus on halting disease progression by reducing inflammation and minimizing additional trauma to the scalp. Common therapies include topical and intralesional corticosteroids, topical calcineurin inhibitors, oral tetracyclines, and systemic agents such as hydroxychloroquine [56, 59].

While the use of light‐based therapy in CCCA is not well established, emerging interest in LLLT has prompted clinical exploration. Specifically, promising results from a dual wavelength LED device have opened a new avenue in the treatment for CCCA. Ongoing clinical trials continue to explore this treatment modality and have shown promising initial results [12]. A case series by Cook and colleagues from 2023 described disease stabilization in addition to both subjective and objective improvement in hair density in four women with CCCA treated with LLLT [60]. More rigorous clinical trials are needed to evaluate the safety and efficacy of light‐based treatment modalities in this form of scarring alopecia.

### 4.7 Traction Alopecia

Traction alopecia (TA) is a form of hair loss caused by prolonged tension on the hair shafts due to certain hairstyles and grooming practices. It is most commonly seen along the frontal and temporal scalp margins and disproportionately affects women of African descent, although it occurs across all ethnic groups [61, 62]. Clinical findings vary depending on the stage of disease: early TA is characterized by perifollicular erythema, follicular hyperkeratosis, and non‐scarring hair loss, whereas chronic TA results in permanent scarring and follicular dropout, particularly at the anterior hairline [63]. The condition is often diagnosed clinically and can be graded using the Marginal Traction Alopecia Severity (M‐TAS) scale, which provides a standardized method for assessing disease severity and progression [61, 64].

The pathogenesis of TA is mechanical in origin, stemming from chronic tension and traction that leads to inflammation, follicular degeneration, and eventually fibrosis if uncorrected. While reversible in early stages, chronic TA can mimic scarring alopecias such as CCCA or FFA [65].

Treatment of TA involves immediate cessation of traction‐inducing hairstyles and patient education on protective styling techniques. Pharmacologic interventions include topical or intralesional corticosteroids to reduce inflammation, topical minoxidil to promote hair regrowth, and in some cases, oral tetracyclines or calcineurin inhibitors when perifollicular inflammation is present [66, 67].

There is limited but emerging evidence that LLLT may play a supportive role in the management of early‐stage TA. LLLT's mechanism of action (see prior sections) could be beneficial if active follicles remain [7].

### 4.8 Scarring Alopecia Secondary to Other Disorders

Secondary scarring alopecia arises from external insults or systemic diseases that destroy the hair follicle and replace it with fibrotic tissue. Unlike primary cicatricial alopecias, which originate from an inflammatory process targeting the follicle itself, secondary scarring alopecias result from broader dermal damage. Common causes include burns, radiation, trauma, certain infections, and autoimmune conditions such as scleroderma and discoid lupus erythematosus (DLE). Regardless of etiology, the destruction of the follicular stem cells and sebaceous glands typically leads to irreversible hair loss [68, 69].

DLE is one of the most frequent autoimmune causes of secondary scarring alopecia. It presents clinically with well‐defined, erythematous plaques featuring scaling, follicular plugging, and central atrophy. Chronic scalp involvement leads to progressive follicular damage and scarring, often with dyspigmentation and telangiectasia in longstanding lesions [68, 70, 71]. Histopathologically, DLE is characterized by interface dermatitis, basement membrane thickening, follicular plugging, and a periadnexal lymphocytic infiltrate [69]. These features contribute to the gradual fibrotic destruction of the follicular epithelium, culminating in permanent alopecia.

Management of all secondary causes of alopecia includes addressing the underlying condition. Specifically, management of DLE centers on reducing inflammation and halting disease progression. Topical corticosteroids and calcineurin inhibitors are first‐line treatments, while systemic therapies may be employed in more severe or recalcitrant cases [72].

Though not widely studied in secondary scarring alopecias, LLLT has shown early promise. A case report described significant clinical improvement in a patient with recalcitrant DLE treated with LLLT, noting reduced erythema and scaling, improved skin texture, and partial hair regrowth [73]. These findings suggest that LLLT may exert anti‐inflammatory and regenerative effects even in settings of autoimmune‐mediated scarring, though larger, broader studies are needed to confirm its role in this population.

## 5 Discussion

This review highlights the growing body of evidence supporting the role of LLLT as adjunctive or primary therapy for a range of alopecia subtypes and hair loss in general. Across the conditions reviewed, LLLT has demonstrated varying degrees of efficacy, with the most robust evidence currently found in the treatment of AGA and AA. For scarring alopecias like FFA and CCCA, evidence is more limited, but results from preliminary clinical trials with dual wavelength LED therapy are promising, and other light‐based therapies may aid in reducing symptoms and stabilizing disease when combined with anti‐inflammatory treatments. In TA and TE, photobiomodulation may support recovery by stimulating follicular activity, though studies remain sparse.

While mechanisms differ by wavelength and device, light‐based therapy generally enhances scalp microcirculation, modulates inflammation, and promotes anagen phase entry. Despite encouraging results, the field is constrained by small sample sizes, inconsistent protocols, and a lack of long‐term data. Additionally, some studies are directly sponsored by device manufacturers, which may present conflicts despite being published in peer‐reviewed journals. Devices such as those that use dual‐wavelength LED light caps show promising results in addition to (and perhaps secondary to) high compliance rates. Given the promising results, clinicians should consider integrating light‐based treatments into individualized regimens, particularly for patients who seek alternatives to systemic therapies or have not responded to conventional treatments.

Future directions should prioritize well‐powered, randomized controlled trials to establish standardized treatment parameters across devices and alopecia types. Studies should explore long‐term outcomes, combination therapies, and biological predictors of response to guide personalized treatment approaches.

## Author Contributions

All authors listed on this manuscript can attest that they have contributed meaningfully to its completion.

## Ethics Statement

The authors have nothing to report.

## Conflicts of Interest

Dr. Mark Nestor is a consultant for Revian.

## Data Availability

The authors have nothing to report.

## References

[1] H. Wolff , T. W. Fischer , and U. Blume‐Peytavi , “The Diagnosis and Treatment of Hair and Scalp Diseases,” Deutsches Ärzteblatt International 113, no. 21 (2016): 377–386, 10.3238/arztebl.2016.0377. 27504707 PMC4908932

[2] N. L. Tamashunas and W. F. Bergfeld , “Male and Female Pattern Hair Loss: Treatable and Worth Treating,” Cleveland Clinic Journal of Medicine 88, no. 3 (2021): 173–182, 10.3949/ccjm.88a.20014. 33648970

[3] A. G. Messenger and R. Sinclair , “Follicular Miniaturization in Female Pattern Hair Loss: Clinicopathological Correlations,” British Journal of Dermatology 155, no. 5 (2006): 926–930, 10.1111/j.1365-2133.2006.07409.x. 17034520

[4] M. S. Nestor , G. Ablon , A. Gade , H. Han , and D. L. Fischer , “Treatment Options for Androgenetic Alopecia: Efficacy, Side Effects, Compliance, Financial Considerations, and Ethics,” Journal of Cosmetic Dermatology 20, no. 12 (2021): 3759–3781, 10.1111/jocd.14537. 34741573 PMC9298335

[5] N. Hunt and S. McHale , “The Psychological Impact of Alopecia,” BMJ 331, no. 7522 (2005): 951–953, 10.1136/bmj.331.7522.951. 16239692 PMC1261195

[6] P. Avci , A. Gupta , M. Sadasivam , et al., “Low‐Level Laser (Light) Therapy (LLLT) in Skin: Stimulating, Healing, Restoring,” Seminars in Cutaneous Medicine and Surgery 32, no. 1 (2013): 41–52. 24049929 PMC4126803

[7] P. Avci , G. K. Gupta , J. Clark , N. Wikonkal , and M. R. Hamblin , “Low‐Level Laser (Light) Therapy (LLLT) for Treatment of Hair Loss,” Lasers in Surgery and Medicine 46, no. 2 (2014): 144–151, 10.1002/lsm.22170. 23970445 PMC3944668

[8] A. K. Gupta and D. Daigle , “The Use of Low‐Level Light Therapy in the Treatment of Androgenetic Alopecia and Female Pattern Hair Loss,” Journal of Dermatological Treatment 25, no. 2 (2014): 162–163, 10.3109/09546634.2013.832134. 23924031

[9] J. J. Jimenez , T. C. Wikramanayake , W. Bergfeld , et al., “Efficacy and Safety of a Low‐Level Laser Device in the Treatment of Male and Female Pattern Hair Loss: A Multicenter, Randomized, Sham Device‐Controlled, Double‐Blind Study,” American Journal of Clinical Dermatology 15, no. 2 (2014): 115–127, 10.1007/s40257-013-0060-6. 24474647 PMC3986893

[10] M. S. Nestor , “Shedding Some Light on Hair Loss,” Practical Dermatology, (2020), accessed April 25, 2025, https://practicaldermatology.com/topics/aesthetics‐cosmeceuticals/shedding‐some‐light‐on‐hair‐loss/23191/.

[11] H. Kim , J. W. Choi , J. Y. Kim , J. W. Shin , S. j. Lee , and C. H. Huh , “Low‐Level Light Therapy for Androgenetic Alopecia: A 24‐Week, Randomized, Double‐Blind, Sham Device–Controlled Multicenter Trial,” Dermatologic Surgery 39, no. 8 (2013): 1177–1183, 10.1111/dsu.12200. 23551662

[12] Wake Forest University Health Sciences , “A Pilot Study of Revian Red All LED Cap as a Novel Treatment for Central Centrifugal Cicatricial Alopecia,” clinicaltrials.gov, (2025), accessed May 1, 2025, https://clinicaltrials.gov/study/NCT04764331.

[13] O. T. Norwood , “Male Pattern Baldness: Classification and Incidence,” Southern Medical Journal 68, no. 11 (1975): 1359–1365, 10.1097/00007611-197511000-00009. 1188424

[14] K. D. Kaufman , “Androgens and Alopecia,” Molecular and Cellular Endocrinology 198, no. 1–2 (2002): 89–95, 10.1016/S0303-7207(02)00372-6. 12573818

[15] R. M. Trüeb , “Molecular Mechanisms of Androgenetic Alopecia,” Experimental Gerontology 37, no. 8–9 (2002): 981–990, 10.1016/S0531-5565(02)00093-1. 12213548

[16] A. Kidangazhiathmana and P. Santhosh , “Pathogenesis of Androgenetic Alopecia,” Clinical Dermatology Review 6, no. 2 (2022): 69–74, 10.4103/cdr.cdr_29_21.

[17] A. Adil and M. Godwin , “The Effectiveness of Treatments for Androgenetic Alopecia: A Systematic Review and Meta‐Analysis,” Journal of the American Academy of Dermatology 77, no. 1 (2017): 136–141.e5, 10.1016/j.jaad.2017.02.054. 28396101

[18] A. K. Gupta and A. Charrette , “Topical Minoxidil: Systematic Review and Meta‐Analysis of Its Efficacy in Androgenetic Alopecia,” Skinmed 13, no. 3 (2015): 185–189. 26380504

[19] J. Shapiro and K. D. Kaufman , “Use of Finasteride in the Treatment of Men With Androgenetic Alopecia (Male Pattern Hair Loss),” Journal of Investigative Dermatology Symposium Proceedings 8, no. 1 (2003): 20–23, 10.1046/j.1523-1747.2003.12167.x. 12894990

[20] R. K. Sivamani , G. Ablon , Y. Nong , J. Maloh , A. Hazan , and I. Raymond , “A Prospective, Multi‐Center Study to Evaluate the Safety and Efficacy of a Vegan Nutraceutical to Improve Hair Growth and Quality in Females Following a Plant‐Based Diet,” Journal of Drugs in Dermatology 23, no. 8 (2024): 661–668, 10.36849/JDD.8421. 39093662

[21] A. K. Gupta , D. C. A. Lyons , and D. Daigle , “Progression of Surgical Hair Restoration Techniques,” Journal of Cutaneous Medicine and Surgery 19, no. 1 (2015): 17–21, 10.2310/7750.2014.13212. 25775658

[22] M. Leavitt , G. Charles , E. Heyman , and D. Michaels , “HairMax LaserComb Laser Phototherapy Device in the Treatment of Male Androgenetic Alopecia: A Randomized, Double‐Blind, Sham Device‐Controlled, Multicentre Trial,” Clinical Drug Investigation 29, no. 5 (2009): 283–292, 10.2165/00044011-200929050-00001. 19366270

[23] J. Kocher , N. Jandick , D. Spragion , et al., “Dual Wavelength LEDs Induce Reactive Oxygen Species and Nitric Oxide That Inhibit the Production of Dihydrotestosterone by 5‐α Reductase,” Journal of Biophotonics 18, no. 2 (2025): e202400388, 10.1002/jbio.202400388. 39667415

[24] M. S. Nestor , B. Berman , R. Sinclair , N. Medendorp , M. Womble , and N. Stasko , “Clinical Efficacy of an At‐Home, 620‐ and 660‐nm Red Light Treatment on Scalp Pruritus and Irritation,” Journal of Clinical and Aesthetic Dermatology 12, no. 5 SUPPL (2020): S21–S22.

[25] M. Thomas , M. Stockslager , J. Oakley , T. M. Womble , and R. Sinclair , “Clinical Safety and Efficacy of Dual Wavelength Low‐Level Light Therapy in Androgenetic Alopecia: A Double‐Blind Randomized Controlled Study,” Dermatologic Surgery 51, no. 4 (2025): 416–421, 10.1097/DSS.0000000000004509. 39679573

[26] R. J. Lanzafame , R. R. Blanche , A. B. Bodian , R. P. Chiacchierini , A. Fernandez‐Obregon , and E. R. Kazmirek , “The Growth of Human Scalp Hair Mediated by Visible Red Light Laser and LED Sources in Males,” Lasers in Surgery and Medicine 45, no. 8 (2013): 487–495, 10.1002/lsm.22173. 24078483

[27] A. Munck , M. F. Gavazzoni , and R. M. Trüeb , “Use of Low‐Level Laser Therapy as Monotherapy or Concomitant Therapy for Male and Female Androgenetic Alopecia,” International Journal of Trichology 6, no. 2 (2014): 45–49, 10.4103/0974-7753.138584. 25191036 PMC4154149

[28] S. Malkud , “Telogen Effluvium: A Review,” Journal of Clinical and Diagnostic Research: JCDR 9, no. 9 (2015): WE01–WE03, 10.7860/JCDR/2015/15219.6492. 26500992 PMC4606321

[29] A. E. Buhl , “Minoxidil's Action in Hair Follicles,” Journal of Investigative Dermatology 96, no. 5 (1991): S73–S74, 10.1111/1523-1747.ep12471911. 2022879

[30] M. Amer , A. Nassar , H. Attallah , and A. Amer , “Results of Low‐Level Laser Therapy in the Treatment of Hair Growth: An Egyptian Experience,” Dermatologic Therapy 34, no. 3 (2021): e14940, 10.1111/dth.14940. 33713522

[31] E. Sorbellini , M. Rucco , and F. Rinaldi , “Photodynamic and Photobiological Effects of Light‐Emitting Diode (LED) Therapy in Dermatological Disease: An Update,” Lasers in Medical Science 33, no. 7 (2018): 1431–1439, 10.1007/s10103-018-2584-8. 30006754 PMC6133043

[32] A. M. Finner , “Alopecia Areata: Clinical Presentation, Diagnosis, and Unusual Cases,” Dermatologic Therapy 24, no. 3 (2011): 348–354, 10.1111/j.1529-8019.2011.01413.x. 21689244

[33] A. Alkhalifah , A. Alsantali , E. Wang , K. J. McElwee , and J. Shapiro , “Alopecia Areata Update: Part I. Clinical Picture, Histopathology, and Pathogenesis,” Journal of the American Academy of Dermatology 62, no. 2 (2010): 177–188, quiz 189–190, 10.1016/j.jaad.2009.10.032. 20115945

[34] A. Gilhar , A. Etzioni , and R. Paus , “Alopecia Areata,” New England Journal of Medicine 366, no. 16 (2012): 1515–1525, 10.1056/NEJMra1103442. 22512484

[35] A. Tosti , S. Bellavista , and M. Iorizzo , “Alopecia Areata: A Long Term Follow‐Up Study of 191 Patients,” Journal of the American Academy of Dermatology 55, no. 3 (2006): 438–441, 10.1016/j.jaad.2006.05.008. 16908349

[36] L. Petukhova , M. Duvic , M. Hordinsky , et al., “Genome‐Wide Association Study in Alopecia Areata Implicates Both Innate and Adaptive Immunity,” Nature 466, no. 7302 (2010): 113–117, 10.1038/nature09114. 20596022 PMC2921172

[37] K. P. Huang , S. Mullangi , Y. Guo , and A. A. Qureshi , “Autoimmune, Atopic, and Mental Health Comorbid Conditions Associated With Alopecia Areata in the United States,” JAMA Dermatology 149, no. 7 (2013): 789–794, 10.1001/jamadermatol.2013.3049. 23700152

[38] B. A. King and B. G. Craiglow , “Janus Kinase Inhibitors for Alopecia Areata,” Journal of the American Academy of Dermatology 89, no. 2S (2023): S29–S32, 10.1016/j.jaad.2023.05.049. 37591562

[39] B. King , X. Zhang , W. G. Harcha , et al., “Efficacy and Safety of Ritlecitinib in Adults and Adolescents With Alopecia Areata: A Randomised, Double‐Blind, Multicentre, Phase 2b‐3 Trial,” Lancet 401, no. 10387 (2023): 1518–1529, 10.1016/S0140-6736(23)00222-2. 37062298

[40] J. M. Bae , B. Y. Hong , J. H. Lee , J. H. Lee , and G. M. Kim , “The Efficacy of 308‐nm Excimer Laser/Light (EL) and Topical Agent Combination Therapy Versus EL Monotherapy for Vitiligo: A Systematic Review and Meta‐Analysis of Randomized Controlled Trials (RCTs),” Journal of the American Academy of Dermatology 74, no. 5 (2016): 907–915, 10.1016/j.jaad.2015.11.044. 26785803

[41] N. Al‐Mutairi , “308‐nm Excimer Laser for the Treatment of Alopecia Areata,” Dermatologic Surgery 33, no. 12 (2007): 1483–1487, 10.1111/j.1524-4725.2007.33320.x. 18076615

[42] M. M. Khalaf and W. A. Abouel‐Naga , “Effect of Polarized Light Therapy on Hair Regrowth in Alopecia Areata,” Medical Journal of Cairo University 86, no. 9 (2018): 2959–2965, 10.21608/mjcu.2018.59859.

[43] T. C. Wikramanayake , R. Rodriguez , S. Choudhary , et al., “Effects of the Lexington LaserComb on Hair Regrowth in the C3H/HeJ Mouse Model of Alopecia Areata,” Lasers in Medical Science 27, no. 2 (2012): 431–436, 10.1007/s10103-011-0953-7. 21739260

[44] K. Svigos , L. Yin , L. Fried , K. Lo Sicco , and J. Shapiro , “A Practical Approach to the Diagnosis and Management of Classic Lichen Planopilaris,” American Journal of Clinical Dermatology 22, no. 5 (2021): 681–692, 10.1007/s40257-021-00630-7. 34347282

[45] H. Kang , A. A. Alzolibani , N. Otberg , and J. Shapiro , “Lichen Planopilaris,” Dermatologic Therapy 21, no. 4 (2008): 249–256, 10.1111/j.1529-8019.2008.00206.x. 18715294

[46] C. O. C. Fechine , N. Y. S. Valente , and R. Romiti , “Lichen Planopilaris and Frontal Fibrosing Alopecia: Review and Update of Diagnostic and Therapeutic Features,” Anais Brasileiros de Dermatologia 97, no. 3 (2022): 348–357, 10.1016/j.abd.2021.08.008. 35379508 PMC9133245

[47] E. Errichetti , M. Figini , M. Croatto , and G. Stinco , “Therapeutic Management of Classic Lichen Planopilaris: A Systematic Review,” Clinical, Cosmetic and Investigational Dermatology 11 (2018): 91–102, 10.2147/CCID.S137870. 29520159 PMC5833781

[48] A. Moussa , B. Bhoyrul , L. Asfour , A. Kazmi , S. Eisman , and R. D. Sinclair , “Treatment of Lichen Planopilaris With Baricitinib: A Retrospective Study,” Journal of the American Academy of Dermatology 87, no. 3 (2022): 663–666, 10.1016/j.jaad.2022.02.027. 35202778

[49] M. Nasimi and M. S. Ansari , “JAK Inhibitors in the Treatment of Lichen Planopilaris,” Skin Appendage Disorders 10, no. 1 (2024): 10–17, 10.1159/000534631. 38313572 PMC10836856

[50] J. Plante , C. Eason , A. Snyder , and D. Elston , “Tofacitinib in the Treatment of Lichen Planopilaris: A Retrospective Review,” Journal of the American Academy of Dermatology 83, no. 5 (2020): 1487–1489, 10.1016/j.jaad.2020.05.104. 32473973

[51] M. J. Randolph , W. A. Salhi , and A. Tosti , “Lichen Planopilaris and Low‐Level Light Therapy: Four Case Reports and Review of the Literature About Low‐Level Light Therapy and Lichenoid Dermatosis,” Dermatologic Therapy 10, no. 2 (2020): 311–319, 10.1007/s13555-020-00359-x. PMC7090134 32060796

[52] S. Vañó‐Galván , A. M. Molina‐Ruiz , C. Serrano‐Falcón , et al., “Frontal Fibrosing Alopecia: A Multicenter Review of 355 Patients,” Journal of the American Academy of Dermatology 70, no. 4 (2014): 670–678, 10.1016/j.jaad.2013.12.003. 24508293

[53] A. Tosti , B. M. Piraccini , M. Iorizzo , and C. Misciali , “Frontal Fibrosing Alopecia in Postmenopausal Women,” Journal of the American Academy of Dermatology 52, no. 1 (2005): 55–60, 10.1016/j.jaad.2004.05.014. 15627081

[54] M. J. Harries , K. Meyer , I. Chaudhry , et al., “Lichen Planopilaris Is Characterized by Immune Privilege Collapse of the Hair Follicle's Epithelial Stem Cell Niche,” Journal of Pathology 231, no. 2 (2013): 236–247, 10.1002/path.4233. 23788005

[55] M. L. Porriño‐Bustamante , M. A. Fernández‐Pugnaire , and S. Arias‐Santiago , “Frontal Fibrosing Alopecia: A Review,” Journal of Clinical Medicine 10, no. 9 (2021): 1805, 10.3390/jcm10091805. 33919069 PMC8122646

[56] T. A. Ogunleye , A. McMichael , and E. A. Olsen , “Central Centrifugal Cicatricial Alopecia: What Has Been Achieved, Current Clues for Future Research,” Dermatologic Clinics 32, no. 2 (2014): 173–181, 10.1016/j.det.2013.12.005. 24680004

[57] C. S. Jordan , C. Chapman , A. Kolivras , J. L. Roberts , N. B. Thompson , and C. T. Thompson , “Clinicopathologic and Immunophenotypic Characterization of Lichen Planopilaris and Central Centrifugal Cicatricial Alopecia: A Comparative Study of 51 Cases,” Journal of Cutaneous Pathology 47, no. 2 (2020): 128–134, 10.1111/cup.13592. 31605498

[58] N. C. Dlova , F. H. Jordaan , O. Sarig , and E. Sprecher , “Autosomal Dominant Inheritance of Central Centrifugal Cicatricial Alopecia in Black South Africans,” Journal of the American Academy of Dermatology 70, no. 4 (2014): 679–682.e1, 10.1016/j.jaad.2013.11.035. 24480456

[59] C. Aguh and A. McMichael , “Central Centrifugal Cicatricial Alopecia,” JAMA Dermatology 156, no. 9 (2020): 1036, 10.1001/jamadermatol.2020.1859. 32745206

[60] M. K. Cook , B. N. Feaster , J. J. Subash , J. Larrondo , and A. J. McMichael , “Use of Low‐Level Light Therapy in Management of Central Centrifugal Cicatricial Alopecia: A Case Series of Four Patients,” Photodermatology, Photoimmunology & Photomedicine 39, no. 6 (2023): 673–675, 10.1111/phpp.12905. 37612840

[61] V. Billero and M. Miteva , “Traction Alopecia: The Root of the Problem,” Clinical, Cosmetic and Investigational Dermatology 11 (2018): 149–159, 10.2147/CCID.S137296. 29670386 PMC5896661

[62] V. D. Callender , A. J. McMichael , and G. F. Cohen , “Medical and Surgical Therapies for Alopecias in Black Women,” Dermatologic Therapy 17, no. 2 (2004): 164–176, 10.1111/j.1396-0296.2004.04017.x. 15113284

[63] A. Samrao , V. H. Price , D. Zedek , and P. Mirmirani , “The “Fringe Sign”—A Useful Clinical Finding in Traction Alopecia of the Marginal Hair Line,” Dermatology Online Journal 17, no. 11 (2011): 1, 10.5070/D325M840MZ. 22136857

[64] N. P. Khumalo , R. M. Ngwanya , S. Jessop , F. Gumedze , and R. Ehrlich , “Marginal Traction Alopecia Severity Score: Development and Test of Reliability,” Journal of Cosmetic Dermatology 6, no. 4 (2007): 262–269, 10.1111/j.1473-2165.2007.00345.x. 18047612

[65] F. Rongioletti and K. Christana , “Cicatricial (Scarring) Alopecias: An Overview of Pathogenesis, Classification, Diagnosis, and Treatment,” American Journal of Clinical Dermatology 13, no. 4 (2012): 247–260, 10.2165/11596960-000000000-00000. 22494477

[66] J. M. Fu and V. H. Price , “Approach to Hair Loss in Women of Color,” Seminars in Cutaneous Medicine and Surgery 28, no. 2 (2009): 109–114, 10.1016/j.sder.2009.04.004. 19608062

[67] J. Raffi , R. Suresh , and O. Agbai , “Clinical Recognition and Management of Alopecia in Women of Color,” International Journal of Women's Dermatology 5, no. 5 (2019): 314–319, 10.1016/j.ijwd.2019.08.005. PMC6938875 31909150

[68] J. C. Achtman and V. P. Werth , “Pathophysiology of Cutaneous Lupus Erythematosus,” Arthritis Research & Therapy 17, no. 1 (2015): 182, 10.1186/s13075-015-0706-2. 26257198 PMC4530484

[69] C. Bernárdez , A. M. Molina‐Ruiz , and L. Requena , “Histologic Features of Alopecias: Part II: Scarring Alopecias,” Actas Dermo‐Sifiliográficas (English Edition) 106, no. 4 (2015): 260–270, 10.1016/j.adengl.2015.03.002. 25439143

[70] N. R. Rowell , “Laboratory Abnormalities in the Diagnosis and Management of Lupus Erythematosus,” British Journal of Dermatology 84, no. 3 (1971): 210–216, 10.1111/j.1365-2133.1971.tb14209.x. 4102091

[71] S. Udompanich , K. Chanprapaph , and P. Suchonwanit , “Hair and Scalp Changes in Cutaneous and Systemic Lupus Erythematosus,” American Journal of Clinical Dermatology 19, no. 5 (2018): 679–694, 10.1007/s40257-018-0363-8. 29948959

[72] S. Jessop , D. A. Whitelaw , and F. M. Delamere , “Drugs for Discoid Lupus Erythematosus,” Cochrane Database of Systematic Reviews 4 (2009): CD002954, 10.1002/14651858.CD002954.pub2. 19821298

[73] N. Kazemikhoo and P. Mansouri , “Successful Treatment of Resistant Discoid Lupus Erythematous (DLE) With Low‐Level Laser Therapy (LLLT): A Case Report,” Journal of Skin and Stem Cell 3, no. 1 (2015): e33145, 10.5812/jssc.33145.

